# Warm-Up and Handgrip Strength in Physically Inactive Chilean Older Females According to Baseline Nutritional Status

**DOI:** 10.3390/ijerph192013335

**Published:** 2022-10-16

**Authors:** Jordan Hernandez-Martinez, María Castillo-Cerda, Tiago Vera-Assaoka, Bastian Carter-Truillier, Tomás Herrera-Valenzuela, Eduardo Guzmán-Muñoz, Braulio Henrique Magnani Branco, Emilio Jofré-Saldía, Pablo Valdés-Badilla

**Affiliations:** 1 Programa de Investigación en Deporte, Sociedad y Buen Vivir, Universidad de los Lagos, Osorno 5290000, Chile; 2 Department of Physical Activity Sciences, Universidad de Los Lagos, Osorno 5290000, Chile; 3 Department of Education, Universidad de Los Lagos, Osorno 5290000, Chile; 4 Faculty of Education, Universidad Católica de Temuco, Temuco 4810302, Chile; 5 Department of Physical Activity, Sports and Health Sciences, Faculty of Medical Sciences, Universidad de Santiago de Chile (USACH), Santiago 8370003, Chile; 6 School of Kinesiology, Faculty of Health, Universidad Santo Tomás, Talca 3530000, Chile; 7 Graduate Program in Health Promotion, Cesumar University (UniCesumar), Maringá 87050-900, Brazil; 8 Facultad de Educación y Ciencias Sociales, Instituto del Deporte y Bienestar, Universidad Andres Bello, Las Condes, Santiago 7550000, Chile; 9 Instituto de Ciencias de la Salud, Universidad de O’Higgins, Rancagua 2820000, Chile; 10 Department of Physical Activity Sciences, Faculty of Education Sciences, Universidad Católica del Maule, Talca 3530000, Chile; 11 Sports Coach Career, School of Education, Universidad Viña del Mar, Viña del Mar 2520000, Chile

**Keywords:** exercise, muscle strength, women, older adults, aging

## Abstract

This study aims to analyze the effect of different types of warm-ups on handgrip strength (HGS) in physically inactive older females. Secondarily, it aims to compare HGS according to their baseline nutritional status. A randomized crossover trial study was conducted with 44 physically inactive older females distributed into normal weight (n = 12, BMI = 23.9 ± 3.2 kg/m^2^), overweight (n =16, BMI = 27 ± 4.7 kg/m^2^) and obese (n = 16, BMI = 31.6 ± 5.3 kg/m^2^), who participated in three warm-up conditions (static stretching condition, SSC; elastic band condition, EBC; and therapeutic compression ball condition, TCBC) and one control condition (CC, no warm-up). All participants performed the four randomized conditions with recovery within 72 h. A significant decrease (*p* < 0.05) in HGS for the dominant and non-dominant hands was observed when comparing SSC vs. CC. In contrast, comparing the warm-up conditions according to the baseline nutritional status, statistically significant differences (*p* < 0.05) were only reported in the obese group in the dominant and non-dominant hand in favor of CC concerning SSC. In conclusion, warm-up with static flexibility led to a decrease in HGS in physically inactive older females. Only the obese group exhibited this result when analyzed by nutritional status.

## 1. Introduction

During aging, there is, among other situations, a decrease in muscle strength that leads to a negative effect on functional capacity and independence, together with an increased risk of mortality, which reaches 60.6% [1,2,3,4]. In addition to the loss of muscle strength, there is a decrease in the volume, frequency, and intensity of physical activity in older people [5]. In Chile, people over 60 years have a high prevalence of physical inactivity that reaches 71%, which increases to 75% for people over 70. Moreover, there is a 76% prevalence of overweight/obesity in the Chilean population over 65 years of age [6].

During aging, there is a decrease in muscle strength (dynapenia) that a decrease reduction may accompany in muscle mass (sarcopenia) and/or an increase in body fat (sarcopenic obesity); both physiological processes occur during old age [7], so it is essential to monitor and detect early alterations in muscle strength [4,8]. In view of this, handgrip strength (HGS) could be a global strength parameter [9]. HGS is a simple test with high reliability and validity to evaluate older people of all socioeconomic groups, nationalities, ethnicities, or cultural groups, which measures the strength exerted by the flexor–extensor muscles of the wrist [8,9]. A decreased HGS leads to a negative effect on functional independence [10,11] and has been associated with an increased risk of depressive symptoms, fall risk, frailty, hospitalizations, functional limitations, cognitive impairment, overweight/obesity, and considering the aspects listed, is related to minor life expectancy [4], promoting a reduced quality of life [12].

In addition, HGS is considered a valuable tool for determining cardiometabolic risk [13] and is regarded as a good predictor of mortality between 20% and 39% for older people [14,15]. In a study conducted by Lera, Albala, Leyton, Márquez, Angel, Saguez and Sánchez [4] in older people (over 60 years of age—the parameter used in countries in development, such as Chile and Brazil), it was found that females with an HGS equal to or less than 15 kg and males with <27 kg had a higher mortality risk of being overweight/obese compared to older people with normal nutritional status. Therefore, it is important to accurately determine the nutritional status [16] during the aging process, as there are changes in body composition such as an increase in body fat percentage, this measure being associated with the determination of nutritional status. This stands in contrast to the body mass index, which does not provide information on the percentage of body fat, which is a relevant element in clinical practice to assessment older people [16]. Likewise, a greater probability (10 times) of having a lower HGS has been reported in older people who have been obese for 30 years or more compared to older people who have been obese recently, with a negative relationship (r = −0.215; *p* = 0.03) between HGS and excess body fat [17].

While the relationship between HGS and the health status (i.e., nutritional status, functionality, depression, cognitive status, mortality) of older people is known [4,10,11,13], there is still no consensus on the effects of warming on HGS in older people [12,14]. For example, the study has shown that warm-up is intended to generate an increase in muscle temperature by facilitating increased blood flow, optimizing metabolic responses, reducing viscous muscle resistance (a smoother contraction) and increasing nerve transmission speed by seeking an optimal muscle temperature range that limits fatigue and maximizes performance [18]. In addition, it has been found that warm-up with muscular endurance or flexibility improves HGS in physically active older people [19]. On the contrary, Hernández-Martínez, et al. [20] detected that warm-up did not lead to improvements in HGS, observing a trend of decreased HGS by static flexibility warm-up; however, such study was conducted with a physically inactive, young female.

Based on the above background, it seems relevant to confirm or refute these results in other populations by exploring the limits of the theories and, ultimately, to help the progress of specific knowledge related to this subject [20,21,22,23]. Taking into account that the type of warm-up can influence HGS as previously described, the main aim of this study was to analyze the effect of different types of warm-ups (static stretching and muscle strength) on HGS in physically inactive older females, and secondarily, to compare HGS according to their baseline nutritional status.

## 2. Materials and Methods

### 2.1. Study Design

**Figure 1:**
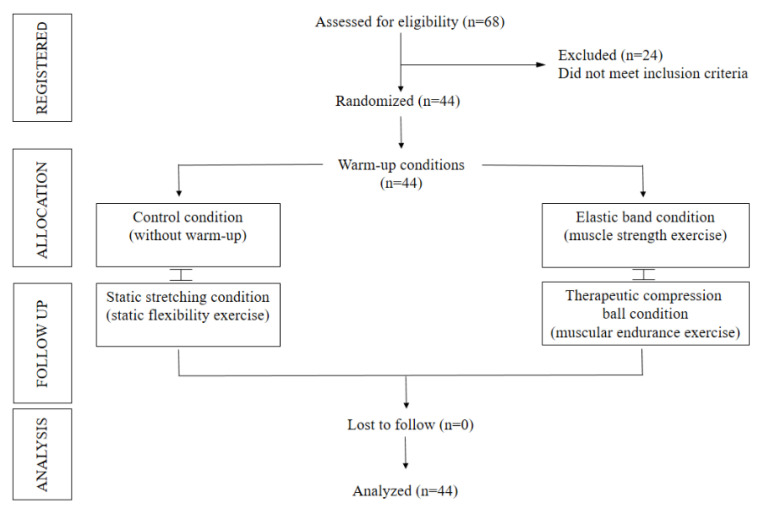
Flowchart of the recruitment process.

**Figure 2:**
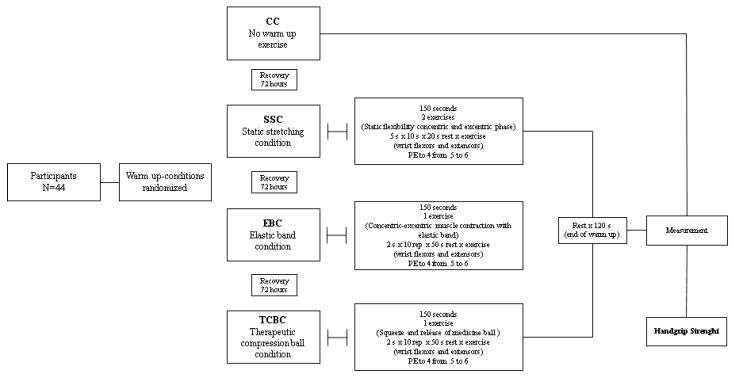
Intervention conditions. Legends: CC: control condition, no warm-up. SSC: static stretching condition, static flexibility exercises. EBC: elastic band condition, muscle strength exercise. TCBC: therapeutic compression ball condition: muscle strength exercise. PE: perceived exertion.

This study is a randomized crossover trial with proportional sampling (https://www.randomizer.org (accessed on 1 January 2022) and homogeneously organized (Figure 1). The variables assessed were HGS in dominant (kg) and non-dominant (kg) hands. A group of older females participated in four intervention conditions (three experimental warm-up conditions and one control condition): (i) control condition (CC) considered no warm-up; (ii) static stretching condition (SSC) consisted of a warm-up by static stretching; (iii) elastic band condition (EBC) was based on a warm-up using an elastic band (THERA BandTM); (iv) therapeutic compression ball condition (TCBC) was a warm-up with the help of a therapeutic compression ball (Ultimate Fitness; weight 0.3 kg; dimensions 9 × 9 cm). All participants performed the four randomized conditions with recovery within 72 h. After each warm-up modality, the HGS assessment was performed and then analyzed according to their baseline nutritional status (see Figure 2). All measurements were performed in the morning (between 9:00 and 11:30 h) and in the same place (Laboratory). The older females did not present pain before performing the warm-up conditions and HGS assessments without presenting musculoskeletal and/or cardiorespiratory injuries during the intervention.

### 2.2. Participants

Forty-four physically inactive older women (normal weight: n = 12; overweight: n = 16; obese: n = 16) were selected by non-probability purposive sampling. Females who did not meet the international recommendations for moderate (<150 to 300 min) or vigorous (<75 to 150 min) physical activity per week were considered physically inactive [24]. The inclusion criteria were: (i) females over 60 years of age; (ii) physically inactive; (iii) physical condition compatible with the practice of physical activity; (iv) ability to understand and follow instructions in a contextualized manner through simple commands. The following were excluded: (i) older females who presented any cardiovascular or respiratory pathology or musculoskeletal injury that prevented them from practicing physical activity; (ii) those who presented moderate or severe cognitive impairment (≥15) assessed by the abbreviated Mini-Mental State Examination [25].

All participants had to accept the criteria for the use and handling of the data by signing an informed consent form authorizing the use of the information for scientific purposes. The research protocol was reviewed and approved by the Scientific Ethics Committee of the Universidad Autónoma de Chile (approval number: 06-2016) and was developed following the guidelines of the Helsinki declaration concerning research involving human subjects.

### 2.3. Morphological Measurements

Each participant’s bipedal height was measured using the Frankfort plane in a horizontal position, with a tape measure (Bodymeter 206, SECA, Hamburg, Germany. Accuracy to 0.1 cm) fixed to the wall. Electrical bioimpedance recording the data in percentage measured body weight and body fat. A bioimpedance apparatus (InBody 120^®^, tetrapolar 8-point tactile electrodes system, model BPM040S12F07, Biospace, Inc., Urbandale, IA, USA) was used, as recommended by the International Society for the Advances of Kinanthropometry (ISAK) [26]. The cut-off points to determine nutritional status according to body fat percentage were based on Macek, et al. [27]: normal weight (26.1 to 33.5%), overweight (33.6 to 39.2%), and obese (>39.2%).

### 2.4. Handgrip Strength (HGS)

HGS was applied to previous recommendations [28]. A sedentary posture was determined as the most appropriate to perform the assessment, including the spine aligned, shoulder in a neutral position, and elbow flexed at 90° to the side of the body, forearm, and wrist in a neutral position. A hand-held dynamometer (Jamar^®^, PLUS+, Sammons Preston Rolyan, Patterson Medical, Chicago, IL, USA). The position of the dynamometer was determined according to the size of the hand allowing a comfortable and functional grip of the instrument with adequate closure of the metacarpal phalangeal and interphalangeal joints, favoring contact between the first phalanx of the index finger and the thumb. Each participant performed three attempts for each hand with a rest of 120 s among each attempt, using the maximum value of the three recorded, which a blinded evaluator recorded. All participants used the same dynamometer model with a neutral position grip.

### 2.5. Intervention Conditions

1. Control Condition (CC): participants remained seated for 3 min before the assessments, then performed the HGS test for the dominant and non-dominant hands.
2. Static Stretching Condition (SSC): participants performed a warm-up with static flexibility for the wrist flexor–extensor muscles, distributed in 5 series of 10 s with a rest of 20 s between each series, executing progressive increases in the amplitude of joint movement, measured using the ten-point Borg’s rating of perceived exertion scale [29] that started between 3 and 5 points and ended between 5 and 6 points. Finally, a 120-s rest was applied to perform the HGS test for the dominant and non-dominant hands.
3. Elastic Band Condition (EBC): participants performed a muscle strength warm-up for the wrist flexor–extensor muscles using an elastic band (THERA BandTM, Akron, OH, USA). Two series of 10 repetitions of 2.5 s per muscle contraction were performed with a rest of 50 s among series, with progressive increases in the tension of the elastic band, which was determined using the OMNI-RES scale (4 to 6 points) [30]. Finally, a 120-s rest was applied to perform the HGS test for the dominant and non-dominant hands.
4. Therapeutic Compression Ball Condition (TCBC): participants performed a muscle strength warm-up for the wrist flexor–extensor muscles using a therapeutic compression ball (Ultimate Fitness; weight 0.3 kg; dimensions 9 × 9 cm), distributed in 2 series of 10 isometric contraction repetitions of 2.5 s each, with a rest of 50 s at the end of the series, with progressive increases in the grip of the therapeutic ball. The intensity started between 3 and 4 points on the Borg’s rating [29] and finished between 5 and 6 points. Finally, a 120-s rest was applied to perform the HGS test for the dominant and non-dominant hands. Figure 2 summarizes the randomized intervention conditions.

### 2.6. Statistical Analysis

Values were reported as mean ± standard deviation. The Shapiro–Wilk test was used to determine the normality of the data, and Levene’s test was used for homogeneity of variance. Normal distribution was observed for all data. To determine the effects and compare the conditions in HGS according to baseline nutritional status, the one-way ANOVA test with Bonferroni’s post hoc was used. The effect size (ES) was calculated with the *d* of Cohen [31], considering a small (0.20–0.49), moderate (0.50–0.79), or strong (>0.80) effect. In all cases, a significance value of *p* < 0.05 was established. The STATISTICA 8 program was used to perform the statistical analysis.

## 3. Results

**Figure 3:**
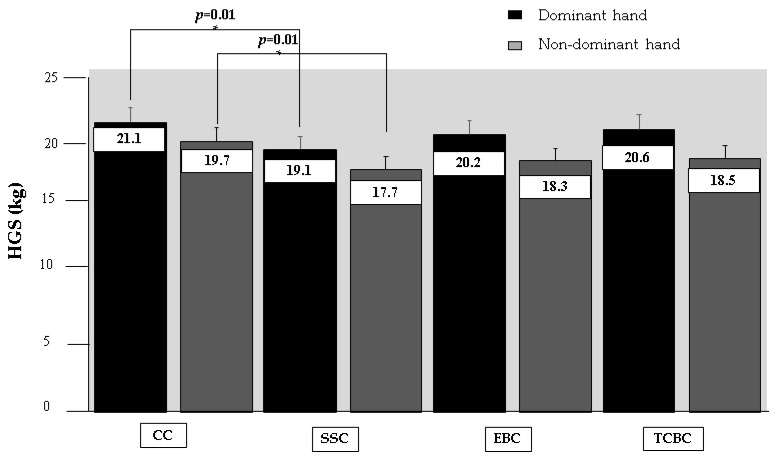
Graphical differences between intervention conditions on handgrip strength. Legends: HGS: handgrip strength. CC: control condition, no warm-up. SSC: static stretching condition, static flexibility exercises. EBC: elastic band condition, muscle strength exercise. TCBC: therapeutic compression ball condition: muscle strength exercise. *: statistically significant differences between conditions.

No significant differences (*p* < 0.05) were found when comparing HGS according to the baseline characteristics of the sample (Table 1). A significant decrease (*p* = 0.01) in HGS for the dominant and non-dominant hands was observed when comparing SSC vs. CC. By contrast, no significant differences were found when comparing EBC, TCBC vs. CC. When comparing the intervention conditions concerning CC, a small to moderate ES was presented with a change rate from 2.3% to 10.1%. These results are presented in Table 2, while Figure 3 synthesizes the HGS comparisons in mean and standard deviation between the intervention conditions.

**Figure 4:**
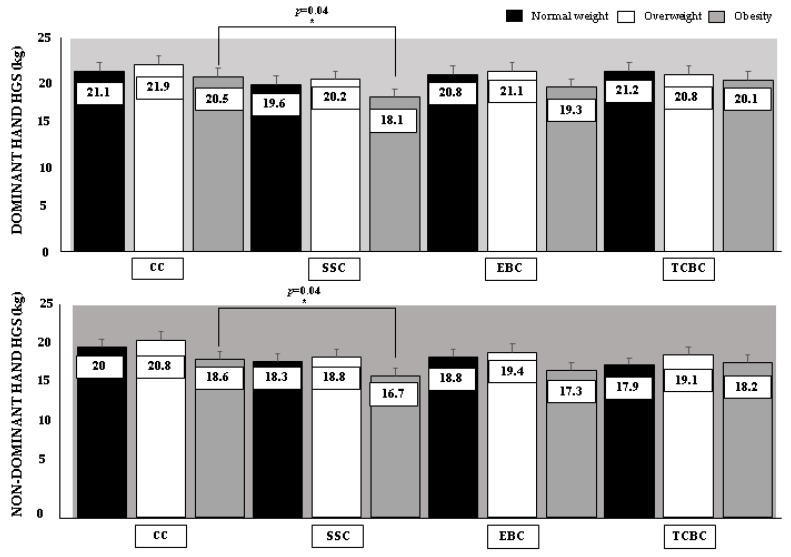
Graphical differences between intervention conditions on handgrip strength according to baseline nutritional status. Legends: HGS: handgrip strength. CC: control condition without warm-up. SSC: static stretching condition, static flexibility exercises. EBC: elastic band condition, muscular endurance exercise. TCBC: therapeutic compression ball condition: muscle strength exercise. *: statistically significant differences between conditions.

When comparing the interventions conditions in HGS according to the baseline nutritional status of the older female, statistically significant differences (*p* < 0.05) were only reported in females with obesity in the dominant and non-dominant hand in favor of CC concerning SSC, whereas no statistically significant differences were detected in overweight and normal weight older females. In addition, a small to moderate ES with a magnitude of change between 0.4% and 11% was presented when comparing all conditions in favor of the CC concerning all warm-up conditions. These results are presented in Table 3, while Figure 4 shows the mean and standard deviation of HGS distributed by intervention conditions and baseline nutritional status.

## 4. Discussion

Among the main results of the study are that the SSC found a significant decrease in HGS for both hands compared to the CC. In addition, when the results were analyzed by baseline nutritional status, only in females with obesity was a decrease in HGS observed when SSC was performed.

HGS during aging begins to decline [10,11]. This loss of muscle strength is associated with alterations in physical independence (e.g., functional capacity) and mental health (e.g., symptoms of depression), leading to a decrease in quality of life and an increased mortality risk in older people [1,12,13,15]. Physically active older people have higher levels of HGS than physically inactive older people. A recent meta-analysis by Ramsey, Rojer, D’Andrea, Otten, Heymans, Trappenburg, Verlaan, Whittaker, Meskers, and Maier [5] reported an association with the lower level of physical activity with decreased HGS in older people (β = 0.041, β = 0.057 and β = 0.070; *p* ≤ 0.001). In the study conducted by Dalleck [32] in physically inactive older people, no significant differences were observed in the Fullerton balance scale when comparing experimental conditions of static (34 points) and dynamic (34.1 points) warm-up concerning the control condition without warm-up (33.1 points). However, in the study of Jung et al. [33], statistically significant differences (*p* < 0.05) were observed in vertical jump height with a decrease of 3.6% by warm-up with static flexibility, 4.4% by neuromuscular facilitation, and 2.6% in the control condition (no warm-up) in adults. In physically inactive youth and adults, it has been shown that a warm-up with static flexibility with a duration ≤60 s leads to a decrease in muscle strength and power of between 1% and 2%, while a duration greater than 60 s leads to decreases of between 4% and 7.5% [34]. The latter antecedents are similar to those detected in the present study, in which SSC led to a decrease in HGS for dominant and non-dominant hands compared to CC reporting a moderate ES (*d* = 0.50) and a magnitude of change located between 9.4% and 10.1%, these differences being statistically significant (*p* = 0.04) in physically inactive older female.

Physical inactivity has been associated with a higher prevalence of overweight/obesity, in addition to increasing the risk of comorbidities in older people, which may affect muscle strength [4,35]. It has been observed that high levels of adiposity can affect agonist muscle activation, which can lead to functional limitations during old age [36]. While it is well known that excess intramuscular fat can affect HGS [37], the scientific literature results do not agree with the impact of warm-up on HGS in overweight females. In the study of Hernández-Martínez, Rauch-Gajardo, Cisterna, Ramírez-Campillo, Moran, Knechtle, Nikolaidis, and Álvarez [20] in overweight youth females, no statistically significant differences were observed between the warm-ups with static flexibility and muscle strength compared to the control condition (no warm-up). However, the findings in our study report a decrease in HGS in obese older females with SSC being a statistically significant difference (*p* < 0.05), presenting a moderate ES (*d* < 0.60) concerning the CC and a magnitude of change between 10.2% and 11%. This indicates that the SSC affects HGS performance in physically inactive older females.

Among the limitations of the study are the following points: (i) not including assessments of neurophysiological mechanisms to determine the activation of the wrist flexor–extensor muscles involved in HGS for the warm-up conditions; (ii) not having physically active older people; however, our study is focused on the characteristics of this population group; (iii) not having males in the sample; (iv) the small sample number, which limited the statistical analyses, and not performing a sample size calculation, which limits the internal consistency and extrapolation of the data; (v) body composition was measured using a standing analyzer, while for older people, due to changes in the thickness of the epidermis and a decrease in the water content in the skin, it is recommended to use electrodes glued directly to the skin; (vi) failure to submit muscle tissue percentage content for the HGS analysis. Among the strengths of the study, we could mention the randomization of the intervention conditions and the analysis of the effects of the warm-up conditions according to baseline nutritional status. Based on the results found in the present study, it can be indicated that the warm-up based on static flexibility led to a decrease in HGS in physically inactive older females, while three isometric tests, without warm-up, allow reaching HGS with high reliability, which is beneficial in the time spent in performing this test, as well as in its applicability in the clinical setting [20]. Future research could delve deeper into exercise characteristics such as volume and intensity that may positively affect HGS in various age and gender ranges.

## 5. Conclusions

Warm-up with static flexibility led to a decrease in HGS in physically inactive older females. Only the obese older female group exhibited this result when an analysis was performed by baseline nutritional status. Therefore, for these older females, it is not recommended to warm-up with static flexibility before performing the HGS assessments.

## Figures and Tables

**Table 1:** Baseline characteristics of the sample.

	Sample (n)	Age (Years)	Body Weight (kg)	Bipedal Height (cm)	BMI (kg/m^2^)	Body Fat (%)	HGS Dominant Hand (kg)	HGS Non-Dominant Hand (kg)
Normal weight	12	68.3 ± 2.1	52.3 ± 2.6	148.1 ± 4.3	23.9 ± 3.2	25.1 ± 5.0	20.6 ± 4.5	18.7 ± 3.3
Overweight	16	71.2 ± 3.5	60.4 ± 3.3	150.5 ± 4.2	27.0 ± 4.7	38.4 ± 7.0	21.0 ± 2.5	19.5 ± 4.1
Obese	16	70.5 ± 2.3	73.1 ± 2.0	152.3 ± 5.0	31.6 ± 5.3	45.2 ± 3.0	19.5 ± 3.6	17.7 ± 2.4

HGS: handgrip strength. n: number. BMI: body mass index.

**Table 2:** Differences between intervention conditions on handgrip strength in physically inactive older females.

Intervention conditions	HGS Dominant Hand (kg)			HGS Non-Dominant Hand (kg)		
	*(d)*	(%)	*(Mean)*	*(d)*	(%)	*(Mean)*
CC vs. SSC	0.50 ^‡^	9.4	*21.1/19.1* *	0.50 ^‡^	10.0	*19.7/17.7* *
CC vs. EBC	0.22 ^†^	4.2	*21.1/20.2*	0.34 ^†^	7.1	*19.7/18.3*
CC vs. TCBC	0.31 ^†^	2.3	21.1/20.6	0.31 ^†^	6.0	19.7/18.5
SSC vs. EBC	0.31 ^†^	5.7	19.1/20.2	0.16 °	3.3	17.7/18.3
SSC vs. TCBC	0.45 ^†^	7.8	19.1/20.6	0.23 ^†^	4.5	17.7/18.5
EBC vs TCBC	0.12 °	1.9	20.2/20.6	0.05 °	1.0	18.3/18.5

HGS: handgrip strength. CC: control condition, without warm-up. SSC: static stretching condition, static flexibility exercises. EBC: elastic band condition, muscle strength exercise. TCBC: therapeutic compression ball condition: muscular endurance exercise. *d*: effect size. °: insignificant effect (<0.20). ^†^: small effect (0.20–0.49). ^‡^: moderate effect (0.50–0.79). *: statistically significant difference.

**Table 3:** Differences between intervention conditions on handgrip strength in physically inactive older females according to their baseline nutritional status.

Intervention Conditions	Normal Weight (n = 12)		Overweight (n = 16)		Obese (n = 16)	
	HGS Dominant Hand (kg)	HGS Non-Dominant Hand (kg)	HGS Dominant Hand (kg)	HGS Non-Dominant Hand (kg)	HGS Dominant Hand (kg)	HGS Non-Dominant Hand (kg)
CC vs. SSC	*d* = 0.29 ^†^, *p* = 0.16 (7.1%)	*d* = 0.34 ^†^, *p* = 0.18 (8.5%)	*d* = 0.54 ^‡^, *p* = 0.09 (10.2%)	*d* = 0.54 ^‡^, *p* = 0.09 (7.7%)	*d* = 0.59 ^‡^, *p* = 0.04 * (11%)	*d* = 0.51 ^‡^, *p* = 0.04 * (10.2%)
CC vs. EBC	*d* = 0.23 ^†^, *p* = 0.13 (1.4%)	*d* = 0.23 ^†^, *p* = 0.15 (6%)	*d* = 0.24 ^†^, *p* = 0.26 (6.8%)	*d* = 0.34 ^†^, *p* = 0.32 (3.6%)	*d* = 0.30 ^†^, *p* = 0.17 (5.8%)	*d* = 0.34 ^†^, *p* = 0.19 (6.8%)
CC vs. TCBC	*d* = 0.44 ^†^, *p* = 0.13 (0.4%)	*d* = 0.44 ^†^, *p* = 0.14 (10.5%)	*d* = 0.34 ^†^, *p* = 0.15 (2.1%)	*d* = 0.34 ^†^, *p* = 0.17 (5%)	*d* = 0.36 ^†^, *p* = 0.30 (2.1%)	*d* = 0.36 ^†^, *p* = 0.36 (1.9%)
SSC vs. EBC	*d* = 0.27 ^†^, *p* = 0.18 (6.1%)	*d* = 0.10 °, *p* = 0.25 (2.7%)	*d* = 0.34 ^†^, *p* = 0.17 (4.4%)	*d* = 0.19 °, *p* = 0.20 (3.1%)	*d* = 0.33 ^†^, *p* = 0.15 (6.6%)	*d* = 0.17 °, *p* = 0.21 (3.5%)
SSC vs. TCBC	*d* = 0.38 ^†^, *p* = 0.21 (8.1%)	*d* = 0.09 °, *p* = 0.21 (2.1%)	*d* = 0.25 ^†^, *p* = 0.16 (2.9%)	*d* = 0.10 °, *p* = 0.28 (1.5%)	*d* = 0.57 ^‡^, *p* = 0.10 (11.4%)	*d* = 0.44 ^†^, *p* = 0.11 (8.9%)
EBC vs. TCBC	*d* = 0.08 °, *p* = 0.17 (1.9%)	*d* = 0.19 °, *p* = 0.17 (4.7%)	*d* = 0.12 °, *p* = 0.21 (1.4%)	*d* = 0.10 °, *p* = 0.21 (1.5%)	*d* = 0.23 ^†^, *p* = 0.15 (4.1%)	*d* = 0.26 ^†^, *p* = 0.15 (5.2%)

HGS: handgrip strength. CC: control condition, without warm-up. SSC: static stretching condition, static flexibility exercises. EBC: elastic band condition, muscle strength exercise. TCBC: therapeutic compression ball condition: muscular endurance exercise. *d*: effect size. °: insignificant effect (<0.20). ^†^: small effect (0.20–0.49). ^‡^: moderate effect (0.50–0.79). *: statistically significant difference.

## Data Availability

The datasets generated during and/or analyzed during the current research are available from the corresponding author on reasonable request.
